# Genome-wide patterns of segregation and linkage disequilibrium: the construction of a linkage genetic map of the poplar rust fungus *Melampsora larici-populina*

**DOI:** 10.3389/fpls.2014.00454

**Published:** 2014-09-10

**Authors:** Michaël Pernaci, Stéphane De Mita, Axelle Andrieux, Jérémy Pétrowski, Fabien Halkett, Sébastien Duplessis, Pascal Frey

**Affiliations:** ^1^Interactions Arbres - Micro organismes, Institut national de la recherche agronomique, UMR1136Champenoux, France; ^2^Interactions Arbres - Micro organismes, Université de Lorraine, UMR1136Vandoeuvre-lès-Nancy, France

**Keywords:** fungal pathogen, linkage mapping, genome mapping, genome sequencing, Mendelian segregation, single-nucleotide polymorphism, selfing, progeny

## Abstract

The poplar rust fungus *Melampsora larici-populina* causes significant yield reduction and severe economic losses in commercial poplar plantations. After several decades of breeding for qualitative resistance and subsequent breakdown of the released resistance genes, breeders now focus on quantitative resistance, perceived to be more durable. But quantitative resistance also can be challenged by an increase of aggressiveness in the pathogen. Thus, it is of primary importance to better understand the genetic architecture of aggressiveness traits. To this aim, our goal is to build a genetic linkage map for *M. larici-populina* in order to map quantitative trait loci related to aggressiveness. First, a large progeny of *M. larici-populina* was generated through selfing of the reference strain 98AG31 (which genome sequence is available) on larch plants, the alternate host of the poplar rust fungus. The progeny's meiotic origin was validated through a segregation analysis of 115 offspring with 14 polymorphic microsatellite markers, of which 12 segregated in the expected 1:2:1 Mendelian ratio. A microsatellite-based linkage disequilibrium analysis allowed us to identify one potential linkage group comprising two scaffolds. The whole genome of a subset of 47 offspring was resequenced using the Illumina HiSeq 2000 technology at a mean sequencing depth of 6X. The reads were mapped onto the reference genome of the parental strain and 144,566 SNPs were identified across the genome. Analysis of distribution and polymorphism of the SNPs along the genome led to the identification of 2580 recombination blocks. A second linkage disequilibrium analysis, using the recombination blocks as markers, allowed us to group 81 scaffolds into 23 potential linkage groups. These preliminary results showed that a high-density linkage map could be constructed by using high-quality SNPs based on low-coverage resequencing of a larger number of *M. larici-populina* offspring.

## Introduction

Poplar is an important product for the wood industry worldwide (Heilman, 1999) and its contribution to energy systems has increased recently (Covarelli et al., 2013). Poplar rust, caused by the pathogenic fungus *Melampsora larici-populina* (Basidiomycota, Pucciniales), is the main phytosanitary constraint for commercial poplar cultivation in Europe and other parts of the world (Gérard et al., 2006; Barrès et al., 2008). In the last 50 years many rust-resistant cultivars were bred and released, but all the qualitative resistance genes (i.e., major resistance genes) released were overcome by pathogen evolution within a short period (Xhaard et al., 2011). Qualitative resistance is particularly subject to breakdown by pathogen evolution for perennial hosts, such as poplar trees, because of the wide inequality between the pathogen's rapid generation time and the time needed to deploy new host varieties (Xu, 2012). Knowledge of the genetic determinism of the virulence factors leading to resistance breakdown would be beneficial both from an academic perspective (e.g., to decipher interactions between avirulence loci and resistance loci, Dangl et al., 2013) and from an applied perspective (e.g., for determining strategies of spatiotemporal management of qualitative resistance, McDonald and Linde, 2002).

The failure of qualitative resistance genes to control poplar rust has prompted poplar breeders to search for quantitative resistance, which is supposed to be more durable (Jorge et al., 2005; Brun et al., 2010; Fabre et al., 2012). Durable resistance is defined as a resistance remaining effective in a cultivar for a long period of time during its widespread cultivation (Johnson, 1979). Nevertheless, quantitative resistance can also be challenged by the evolution of aggressiveness, which is the quantitative component of pathogenicity, determined by several disease-associated traits (Andrivon et al., 2007; Pariaud et al., 2009; Dowkiw et al., 2010). Thus, it is of primary importance to assess the potential evolution of aggressiveness traits in the pathogen and the potential trade-offs between these traits (Lannou, 2012). Knowledge of the genetic determinism of such aggressiveness life history traits (latency period, infection efficiency, sporulation capacity, lesion size, etc.) would be useful in order to guide breeding strategies toward durable resistance. To this aim, a genetic linkage map of the poplar rust fungus would allow examination of the genetic architecture of those traits and mapping the QTLs related to aggressiveness.

The genome of *M. larici-populina* was shotgun sequenced at a coverage of ~6.9X, assembled and annotated recently by the US Department of Energy Joint Genome Institute and an international consortium (Duplessis et al., 2011). The 101.1 Mbp genome is assembled into 462 scaffolds and contains a total of 16,399 predicted gene models. About half of the genome is contained in 27 scaffolds all at least 1.1 Mbp in length. Therefore, the construction of a genetic map of *M. larici-populina* and its integration with the physical map would enable us to identify a chromosomal order of scaffolds and provide a valuable resource for fine mapping and positional cloning of QTLs associated with aggressiveness traits (Hahn et al., 2014).

Compared to plant and animal models, there has been much less interest in genetic mapping for fungi (for a review, see Foulongne-Oriol, 2012) and Oomycetes (Sicard et al., 2003). Among Ascomycota, genetic linkage maps have been developed for some major pathogens of economically important crops, such as *Blumeria graminis* (Pedersen et al., 2002), *Magnaporthe oryzae* (Kaye et al., 2003), *Leptosphaeria maculans* (Cozijnsen et al., 2000), *Mycosphaerella graminicola* (Kema et al., 2002), *Mycosphaerella fijiensis* (Manzo-Sánchez et al., 2008), and *Venturia inaequalis* (Broggini et al., 2011). Among Basidiomycota, most of the mapping efforts have been devoted to economically important edible mushrooms, such as *Agaricus bisporus* (Foulongne-Oriol et al., 2010), *Lentinula edodes* (Terashima et al., 2002), and *Pleurotus ostreatus* (Larraya et al., 2000), and some model ectomycorrhizal fungi (Doudrick et al., 1995; Labbé et al., 2008). Obtaining controlled crosses (either selfed or outcrossed) is even more challenging for rust fungi (Pucciniales), since (i) they are obligate biotrophs, which precludes crosses to be made *in vitro*, and (ii) most of the rust fungi are heteroecious, thus the completion of sexual crosses requires two non-related host plants (Leonard and Szabo, 2005). As a result there are very few reports of genetic linkage maps in rust fungi. To our knowledge, partial genetic maps have been built only for the fusiform rust fungus, *Cronartium quercuum* f. sp. *fusiforme* (Doudrick et al., 1993; Kubisiak et al., 2011), and the wheat stem rust fungus, *Puccinia graminis* f. sp. *tritici* (Zambino et al., 2000). A genetic linkage map is also being constructed for the wheat leaf rust fungus, *Puccinia triticina* (Duplessis et al., 2014).

Genetic mapping is primarily based on the genotyping of a large number of individuals from a controlled cross, using PCR-based (SSR, AFLP, SNP, etc.) molecular markers. For a genetic map of sufficient density, the development of a large number of markers and the genotyping of a large number of progeny represent significant costs, both in time and in money. With the advent of next generation sequencing (NGS) techniques, combined with genome-wide marker discovery techniques such as reduced-representation sequencing (RRS), restriction-site-associated DNA sequencing (RAD-seq) or multiplexed shotgun genotyping (MSG), it is now possible to overcome the technical and financial constraints of genetic mapping (Davey et al., 2011). New technologies for high throughput sequencing, such as Illumina sequencing, open the way to new genotyping and genetic mapping strategies based on re-sequencing at low coverage of a large number of progeny (Huang et al., 2009; Xie et al., 2010). In addition, tagging techniques for multiplex sequencing of numerous individuals on a single Illumina sequencing lane further reduces the cost of re-sequencing (Cronn et al., 2008). This new approach has been successfully applied to build a ultra-high density genetic map of rice, through sequencing of 150 Recombinant Inbred Lines (RILs) to a depth of 0.02X (Huang et al., 2009). This new methodology was found about 20 times faster in data collection, and the linkage map obtained was 35 times more precise in recombination breakpoint determination, compared to the use of PCR-based markers. The accuracy of QTL detection was also improved (Huang et al., 2009; Xie et al., 2010; Yu et al., 2011). This new genetic mapping strategy has been recently applied to plants (Huang et al., 2012; Zhou et al., 2014) and animals (Andolfatto et al., 2011; You et al., 2013). It was also used for the first time to build a high-density sequence-based genetic linkage map for a fungus, the Shiitake mushroom, *L. edodes* (Au et al., 2013). This example provides a proof-of-principle that low-coverage resequencing could allow rapid genotyping of basidiospore-derived progenies, thus facilitating the construction of high-density genetic linkage maps of Basidiomycota for QTL mapping (e.g., of aggressiveness traits in the case of phytopathogenic fungi) and improvement of whole-genome assembly.

The purpose of this work was (i) to generate S1 progeny of *M. larici-populina* through selfing of the reference strain 98AG31 on larch plants; (ii) to characterize the S1 progeny through a segregation analysis of polymorphic microsatellite loci; (iii) to test for linkage disequilibrium between these loci in the progeny; and (iv) to perform a pilot study of genetic mapping through whole-genome resequencing of a subset of 47 S1 individuals.

## Materials and methods

### Production of the *M. larici-populina* S1 progeny

Since *M. larici-populina* is a heteroecious and macrocyclic rust fungus, the completion of the life cycle requires two unrelated host plants, poplar and larch [for detailed life cycles of *Melampsora* spp. and *M. larici-populina* see Vialle et al. (2011) and Hacquard et al. (2011), respectively].

#### Production of M. larici-populina telia

Poplar plants (*Populus deltoides* × *P. nigra* ‘Robusta’) were grown from dormant cuttings in 5-l pots containing a sand-peat (50:50, v/v) mixture, with an initial fertilization of 3.5 g.l^−1^ CaCO_3_ and 6 g.l^−1^ of slow release 13:13:13 N:P:K fertilizer (Nutricote T 100). The plants were grown for 4 months (June–September) in a non-heated greenhouse with natural photoperiod and were watered daily with deionized water. After 4 months, young trees were about 1.2 m high and exhibited 25–30 fully expanded leaves. In late September, 20 poplar plants were spray-inoculated by *M. larici-populina* 98AG31. A urediniospore suspension (40,000 urediniospores.ml^−1^) was sprayed (ca. 1.5 ml per leaf) on the abaxial (lower) surface of each fully expanded leaf with a fine atomization paint sprayer (Pico-Bel, Wagner, Germany). The inoculated plants were maintained under plastic bags overnight in order to ensure 100% relative humidity during the first steps of the infection process (Pinon et al., 2006). After 1 day, the plastic bags were removed and replaced by bags made of cotton fine mesh cloth (porosity < 20 μm), in order to avoid dissemination of rust urediniospores in the greenhouse. The plants were maintained in the non-heated greenhouse throughout autumn (September–December) in order to induce the formation of telia during autumnal senescence of the plants. They were visually inspected every week to check the formation of uredinia and subsequently telia on the leaves (Hacquard et al., 2013). After 3 months, the fallen poplar leaves were collected. Leaves bearing telia were placed in 50 × 20 cm bags made of 1 cm plastic mesh. The bags were allowed to sit on the soil outdoors during a continental European winter (December–February) in order to break teliospore dormancy through natural alternation of freezing/thawing and wetting/drying (Leonard and Szabo, 2005). Starting in February, one leaf was sampled every week to assess whether teliospores would germinate at 19 ± 1°C (see below). Once teliospore dormancy was broken (2–3 months), poplar leaves were collected and stored in a refrigerator (7 ± 1°C) until used for larch inoculation.

#### Inoculation of larch plants

Larch (*Larix decidua*) seedlings were grown in spring in small (8 × 12 × 5 cm) plastic containers in a greenhouse. When they were 5–8 cm tall, larch plants were inoculated with *M. larici-populina* basidiospores. For this, poplar leaves bearing telia were soaked in tap water for 6 h, and then incubated at 19 ± 1°C, adaxial (upper) surface uppermost, on wet filter paper in large (24 × 24 cm) Petri dishes. After 24 h, the production of basidia and basidiospores on the poplar leaves was checked under a stereomicroscope. Leaves producing large quantities of basidiospores were placed 5 cm over larch seedlings for 1 day, in large (30 × 20 × 15 cm) transparent plastic boxes (Curver). After 1 day, the poplar leaves were withdrawn, and the larch seedlings were maintained in the plastic boxes in a growth chamber (19 ± 1°C, photoperiod 16/8 h) for 4–5 weeks. The larch plants were visually inspected every day to check the formation of pycnia, i.e., the haploid stage of the rust fungus, and subsequently aecia on the larch needles. Pycnia were not manually crossed, but spermatization of pycnia occurred naturally, likely through contacts between larch needles, or through the presence of small flies (dark-winged fungus gnats, Sciaridae, Diptera) that thrive in the larch seedling substratum.

#### Production of urediniospores of the offspring

Larch needles bearing individual aecia were harvested every 2 days, and then aeciospores from each single aecium (i.e., S1 individual) were inoculated onto one 12-mm-diameter poplar leaf disc (*Populus deltoides* × *P. nigra* ‘Robusta’) as previously described (Husson et al., 2013). After 8–10 days incubation, the sporulating leaf discs were used to inoculate 4–5 entire poplar leaves as previously described (Husson et al., 2013), in order to obtain 50–100 mg of clonal urediniospores per S1 individual for genomic DNA extraction.

### Genetic analysis

#### Genotyping of the offspring

In order to avoid any bias in segregation and linkage disequilibrium analyses, the genetic purity of 138 of the offspring was verified twice through genotyping with a set of 25 polymorphic microsatellite loci specific to *M. larici-populina*: MLP12 (Barrès et al., 2006), MLP49, MLP50, MLP54, MLP55, MLP56, MLP57, MLP58, MLP59, MLP66, MLP68, MLP71, MLP73, MLP77, MLP82, MLP83, MLP87, MLP91, MLP92, MLP93, MLP94, MLP95, MLP96, MLP97, and MLP100 (Xhaard et al., 2009, 2011). The first verification was performed on the poplar leaf disc inoculated with aeciospores from larch, in order to check the presence of a unique genotype in each S1 individual. The second verification was performed on the urediniospores after multiplication on poplar leaves, in order to detect any contamination from a non-related rust isolate. DNA was extracted from infected poplar leaf discs and from urediniospores using the BioSprint 96 DNA plant kit used in combination with the BioSprint automated workstation (Qiagen), as previously described (Barrès et al., 2008). Microsatellite loci amplification and fragment analysis were performed as previously described (Xhaard et al., 2011).

#### Segregation and linkage disequilibrium analyses

Segregation and linkage disequilibrium analyses were performed on 115 genetically pure S1 individuals, using the 14 microsatellite loci (out of the 25), which are heterozygous in the parental strain 98AG31 (Table 1). According to the Mendelian laws, loci which are homozygous in the parental strain are not expected to segregate in the progeny, whereas a 1 (homozygous 1): 2 (heterozygous): 1 (homozygous 2) segregation is expected for loci which are heterozygous in the parental strain. Chi-squared tests with a significance level of 0.05 were performed to test whether the observed segregation deviated from the expected ratio.

**Table 1:** **Characteristics and genome position of the 14 microsatellite loci, polymorphic in strain 98AG31, used for segregation and linkage disequilibrium analyses in *M. larici-populina***.

**Locus name**	**Repeat motif**	**Scaffold no**.	**Position (bp)**	**Scaffold length (bp)**
MLP49	(GAT)18	37	803,561	929,451
MLP54	(ATG)14	8	807,623	2,004,758
MLP55	(ATC)15	1	2,804,879	4,071,029
MLP56	(AAC)7	5	2,550,898	2,603,268
MLP58	(AAG)12	3	728,772	3,255,379
MLP59	(ATC)13	8	1,544,938	2,004,758
MLP73	(AC)14	38	809,210	903,405
MLP77	(AT)10	15	629,856	1,649,323
MLP82	(TAC)10	15	1,199,792	1,649,323
MLP87	(TGT)8	11	834,087	1,841,903
MLP91	(GTT)10	40	444,668	887,115
MLP92	(TTG)11	26	241,476	1,146,214
MLP94	(TTC)10	1	236,499	4,071,029
MLP12	(AAG)10	NA	NA	NA

Scaffold/sequence position is given according to the M. larici-populina genome sequence v1.0 (http://genome.jgi-psf.org/Mellp1) retrieved on April 1, 2014. NA, not available.

Linkage disequilibrium between all pairs of loci was tested using Fisher's exact test procedure (Garnier-Gere and Dillmann, 1992) implemented in GENEPOP on the Web (http://genepop.curtin.edu.au) (Rousset, 2008) with the following parameters of the Markov chain: 2000 dememorization steps, 250 batches, and 2000 iterations per batch. To adjust the resulting *P*-value distribution for multiple tests, we used the false discovery rate (FDR) procedure (Benjamini and Yekutieli, 2001). The resulting adjusted *P*-values are called *Q*-values. This procedure is implemented in the R package *Q*-value (Storey and Tibshirani, 2003).

### Genomic analysis

#### Genomic DNA extraction

Genomic DNA was extracted from a subset of 47 genetically pure S1 individuals of *M. larici-populina*, plus the parental strain 98AG31. For each individual, 10 aliquots of 5 mg of urediniospores were placed in 2 ml Eppendorf tube, with two 3-mm-diameter glass beads and 20 1-mm-diameter glass beads. The spores were homogenized for 1 min at 30 Hz using a MM 200 Mixer Mill (TissueLyser, Retsch, Qiagen) and then suspended in 1 ml of hot (65°C) CTAB buffer (CTAB 2%, Tris pH9 0.1 M, NaCl 1.4 M, EDTA 0.02 M, β-mercaptoethanol 0.2%). The content of the 10 Eppendorf tubes was pooled in a 50 ml Falcon tube. After carefully mixing by inverting, the tubes were incubated for 30 min at 65°C. One volume of phenol/chloroform/isoamyl alcohol (50:48:2) (Euromedex) was added to the 50 ml Falcon tube. The content of the tube was carefully mixed and then centrifuged at 8000 rpm for 10 min. The aqueous phase was transferred to a new tube and one volume of chloroform was added. The content of the tube was carefully mixed and then centrifuged at 8000 rpm for 10 min. The aqueous phase was transferred to a new tube. RNA was digested with 100 μl of RNaseA (Fermentas, 10 μg.μl^−1^) for 30 min at 37°C. One volume of chloroform was added and the tubes were centrifuged at 8000 rpm for 10 min. The aqueous phase was recovered, distributed as aliquots of 800 μl in Eppendorf tubes, and 600 μl of isopropanol was added to each tube. Then the tubes were centrifuged at 14,000 rpm for 30 min at 4°C. The supernatant was removed by pouring liquid from the tube, and the DNA pellets were washed with 200 μl of 70% ethanol. The tubes were centrifuged at 14,000 rpm for 10 min at 4°C and washed again with 70 μl of 70% ethanol. The supernatant was removed by pipetting liquid from the tubes, the pellets were dried for 30 min under a fume hood, and then resuspended in TE 1× buffer (Tris 10 mM, EDTA 1 mM, pH 8.0). Quality and quantity of recovered high molecular weight DNA was assessed by electrophoresis on agarose gel and with a QuBit fluorometer (Life Technologies).

#### Whole-genome resequencing

Genome DNA re-sequencing was performed by IntegraGen (Evry, France). Forty-eight genomic DNA libraries were prepared using TruSeq DNA sample preparation kit (v3) followed by paired-end 100 bases massively parallel sequencing on Illumina HiSeq 2000. Briefly, 3 μg of each sample of genomic DNA were fragmented by sonication and purified to yield fragments of 400–500 bp. Paired-end adaptor oligonucleotides from Illumina were ligated on repaired A-tailed DNA fragments, then purified and enriched by PCR cycles. Each library was quantified by qPCR before equimolar pooling of the 48 libraries. The 48-plex pool was sequenced on one flowcell lane of Illumina HiSeq 2000 platform as paired-end 100 bp reads. Image analysis and base calling were performed using Illumina Real Time Analysis (RTA) Pipeline with default parameters.

#### Mapping and SNP detection

Mapping was performed with BWA version 0.6.2 (Li and Durbin, 2009). Version 1.0 of the *M. larici-populina* 98AG31 genome assembly (http://genome.jgi-psf.org/Mellp1) was used as reference in index using the IS algorithm. The reference genome contains 462 scaffolds and a total of 101,129,028 bp. Read alignment was performed using default options of the *aln* and *sampe* commands except maximum insert size (750 bp). Alignments were stored in the pileup format using SAMtools version 0.1.18 (Li et al., 2009).

We filtered sites presenting a potential single-nucleotide polymorphism (SNP) using liberal thresholds. All 48 individuals (including the parental strain) were considered jointly for each position, excluding all reads for which the base at this position was called with a quality Phred score lower than 25. We considered sites for which the minority allele (the second in frequency, if more than two) was represented by at least three copies, when merging all individuals. It was also required that each of the three genotypes was represented by two individuals each, where individuals were crudely assigned to one of the three genotypes (heterozygote if both alleles were represented, homozygote otherwise). If more than two alleles were observed, only the two most frequent were considered.

#### Identification of recombination blocks

In order to take into account genotype uncertainty when sequencing depth for a single individual is low, we used an *ad-hoc* scoring function to represent the amount of evidence regarding the homozygous/heterozygous status of each site. The function gives a score of 0 (undetermined) if the depth is 0 or 1, positive scores if only one allele is observed and negative scores if two alleles are observed. Overall, scores are bounded by −1 and +1. We fitted the logistic function *c*/(1 + exp[–*r*(*x* – *b*)]), where *x* is the sequencing depth at a given SNP, to the observed proportion of observed heterozygotes in the 98AG31 parental strain, which is necessarily heterozygous in all true SNP positions. Minimum mean square error estimation yielded *r* = 0.51714, *b* = 3.9148, and *c* = 0.98404. This function was assumed to give the probability of observing a heterozygote if the genotype is truly heterozygous. Since, in our setting, true heterozygotes and true homozygotes are equally likely, we can use the same function as the probability that a genotype is truly homozygous if it is observed to be homozygous. If a genotype was observed to be homozygous, we used an arbitrary weighting scheme to take into account both depth and allele frequency evenness when considering genotypes observed to be heterozygous. The score was computed as (*M*/*m*-1)/200-1 where *M* and *m* are the majority and minority allele absolute frequencies, respectively. In addition, the score of each SNP incorporates the scores of neighboring SNPs. Thus, the score of a given position was actually computed as the sum of the score of the focus SNP and the 15 neighboring SNPs on each side, weighted by the distance (a normal distribution with standard deviation 10,000 bp is used for weights, which gives high weights to SNPs about 20,000 kb apart).

A further step of smoothing was performed in order to identify recombination blocks. We assumed that a region of consecutive SNPs with constant heterozygous or homozygous status to be a block that was transmitted without recombination. We defined these blocks as regions of consecutive SNPs for which the score kept the same sign and at least one SNP exhibited an absolute value larger than 0.5. To avoid excessive false positive recombination points, we extended recombination blocks over SNPs with scores of opposite sign provided that they did not exceed |0.2| and up to a SNP with a score of at least |0.2| of the corresponding sign. These blocks represented overlapping regions of the genome with different putative recombination points defined in each individual. We defined a sample-wise set of blocks by dividing the genome in regions based on all putative recombination points. As a result recombination blocks were regions in which no recombination event had taken place.

Next, we generated the phased sequence of the two parental haplotypes of all blocks. We retrieved the majority allele of all SNPs for individuals that were classified as homozygous for the region under consideration. This generated the sequence of all putative homozygotes for each non-recombining region. We generated the maximum-likelihood phylogeny of those sequences using PhyML version 20120412 (Guindon and Gascuel, 2003) using the Jukes and Cantor model of substitution (all other parameters left to defaults). The two alleles should be represented by two deeply diverged clades in the resulting tree. For this reason, we excluded all recombination groups for which there was no internal branch representing at least 90% of the total tree length. This is expected to exclude recombination groups that contained undetected recombination events or a large proportion of erroneous data.

For all pairs of recombination blocks, we used *P*-value for Fisher's exact test of independence (based on the 3 × 3 matrix of the three genotype frequencies at both sites) as a measure of linkage disequilibrium. The *P*-value was not computed if fewer than 10 individuals had non-missing data for both sites, or if there was less than one copy of each homozygous genotype and fewer than two copies of each heterozygous genotype (over the two loci).

## Results

### Production of the *M. larici-populina* S1 progeny

**Figure 1:**
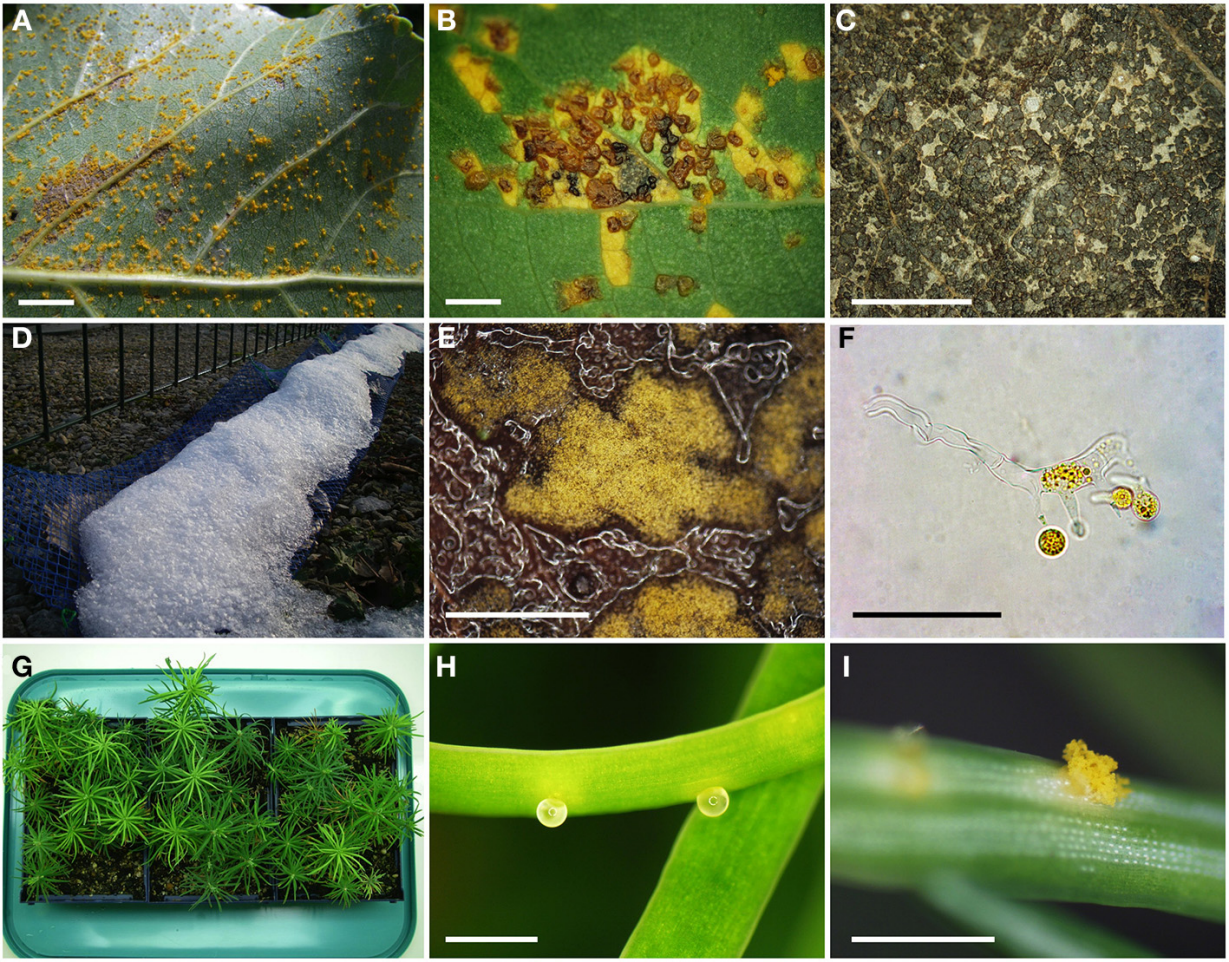
**Successive stages of the life cycle observed in the production of the *M. larici-populina* S1 progeny. (A)** Inoculated poplar leaf covered with uredinia on the abaxial surface (scale bar 1 cm). **(B)** Light brown to dark brown telia forming on the adaxial surface of a poplar leaf (scale bar 1 mm). **(C)** Black mature telia obtained at leaf fall (scale bar 5 mm). **(D)** Outdoor overwintering of poplar leaves in the plastic mesh bags. **(E)** Telia producing basidia and basidiospores (scale bar 1 mm). **(F)** Basidium producing four basidiospores (scale bar 50 μm). **(G)** Young larch seedlings used for inoculation with basidiospores. **(H)** Pycnia on the adaxial surface of a larch needle (scale bar 1 mm). **(I)** Aecium producing aeciospores on the abaxial surface of a larch needle (scale bar 1 mm).

In order to generate the *M. larici-populina* S1 progeny, 4-month-old poplar plants were spray-inoculated with *M. larici-populina* strain 98AG31 in a greenhouse. Uredinia appeared on the abaxial surface of the inoculated poplar leaves 8–10 days after inoculation (Figure 1A). Pale brown telia began to appear on the adaxial surface of the leaves 3–4 weeks after inoculation (Figure 1B) and became dark brown and then black during the following weeks, as the leaves became senescent (Figure 1C). In December, almost all the poplar leaves were covered with black telia. After overwintering for 2 months outside in natural winter conditions (Figure 1D), poplar leaves bearing telia were tested for basidiospores production in laboratory. After water-soaking and incubation on wet filter paper for 1 day, masses of basidia and basidiospores, resulting from meiosis, were produced on the adaxial surface of the leaves (Figures 1E,F). Basidia were used to inoculate larch seedlings (Figure 1G). Pycnia appeared on the adaxial surface of larch needles 5–7 days after inoculation (Figure 1H). Spermatization of pycnia by pycniospores of the opposite mating type occurred naturally, resulting in plasmogamy of two haploid cells and formation of a dikaryotic mycelium. The resulting aecia appeared on the abaxial surface of larch needles 10–15 days after inoculation (Figure 1I), and individual aecia representing single selfed progeny were harvested and then multiplied on poplar leaf discs.

### Genotyping of the offspring

The genetic purity of 138 of the offspring was verified twice (on initial inoculation onto poplar leaf discs and after 2–3 rounds of multiplication on poplar leaves) through genotyping with a set of 25 polymorphic microsatellite loci specific to *M. larici-populina*. Results of genotyping allowed the detection of two types of contamination that may occur during the offspring production process. On the one hand, 4.3% of the individuals exhibited an “external” contamination, i.e., a contamination of an offspring with a non-related *M. larici-populina* isolate, resulting in the presence of non-parental alleles at one or several microsatellite loci. On the other hand, 12.3% of the individuals exhibited an “internal” contamination, i.e., a mixture of two of the offspring, resulting in the presence of unbalanced peak heights of parental alleles at one or several heterozygous microsatellite loci. Both types of contamination were detected at the first (i.e., poplar leaf disc inoculated with aeciospores from larch) and the second (i.e., urediniospores after multiplication on poplar leaves) verification. Isolates identified as contaminated were deleted from further analyses.

### Segregation analysis

**Figure 2:**
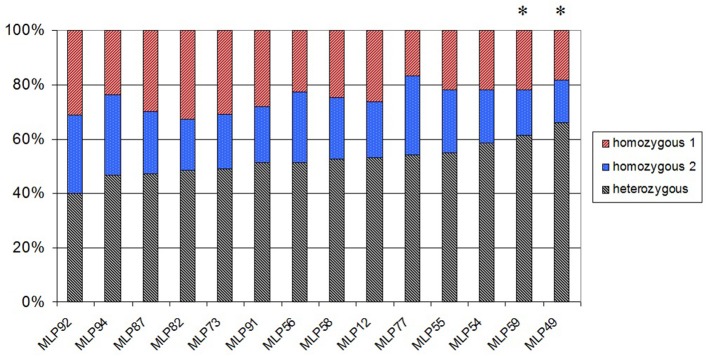
**Percentage of individuals in the *M. larici-populina* S1 progeny, which are heterozygous, homozygous 1, and homozygous 2 at each of the 14 polymorphic microsatellite loci**. Stars denote loci with a significant deviation from the expected 1:2:1 Mendelian segregation for α = 0.05.

In order to validate the progeny's meiotic origin, a segregation analysis was performed on 115 genetically pure S1 individuals, using the 14 microsatellite loci (out of the 25), which are heterozygous in the parental strain 98AG31. Twelve out of the 14 microsatellite loci exhibited a Mendelian segregation of 2 (heterozygous): 1 (homozygous 1): 1 (homozygous 2), as expected (Figure 2). Two loci (MLP59 and MLP49) differed significantly from the expected ratio (*P*-value = 0.034 and 0.005, respectively), exhibiting an excess of heterozygotes (61.4 and 65.8% of heterozygotes, respectively).

### Linkage disequilibrium analysis

In order to detect pairs of microsatellite loci, which are genetically linked, a linkage disequilibrium analysis was performed between all pairs of microsatellite loci. Two out of the 91 pairwise linkage disequilibrium tests were found significant (*Q*-value < 0.05) (Table 2). Highly significant linkage disequilibrium was found for MLP77/MLP56 and MLP77/MLP82 pairs, with *Q*-value < 10^−4^ for both. Therefore, these two pairs of loci are genetically linked pairwise. Conversely, no significant linkage disequilibrium was found for all other pairs of loci, with *Q*-value ranging from 0.253 to 0.808.

**Table 2:** **Matrix of *Q*-values for all pairwise linkage disequilibrium tests between microsatellite loci**.

	**MLP12**	**MLP49**	**MLP54**	**MLP55**	**MLP56**	**MLP58**	**MLP59**	**MLP73**	**MLP77**	**MLP82**	**MLP87**	**MLP94**	**MLP92**
MLP49	0.73544												
MLP54	0.69846	0.73544											
MLP55	0.73544	0.73544	0.73544										
MLP56	0.73544	0.73544	0.73544	0.73544									
MLP58	0.73544	0.34450	0.78528	0.73544	0.73544								
MLP59	0.57010	0.57010	0.73544	0.73544	0.78528	0.38876							
MLP73	0.73544	0.69500	0.73544	0.73544	0.73544	0.78528	0.34450						
MLP77	0.80770	0.73544	0.57010	0.80770	**<10^−4^**	0.52127	0.52127	0.78670					
MLP82	0.78399	0.73544	0.52127	0.73544	0.73544	0.76627	0.73544	0.76627	**<10^−4^**				
MLP87	0.73544	0.73544	0.73544	0.69846	0.73544	0.76627	0.78528	0.73544	0.76627	0.76627			
MLP94	0.69846	0.73544	0.39876	0.77008	0.78528	0.73544	0.73544	0.80770	0.80770	0.76627	0.76627		
MLP92	0.73544	0.73544	0.80770	0.73544	0.78527	0.57010	0.44613	0.73544	0.73544	0.34450	0.73544	0.76627	
MLP91	0.73544	0.44613	0.78399	0.58058	0.73544	0.25345	0.30801	0.73544	0.39876	0.39876	0.73544	0.73544	0.73544

Significant Q-values (Q < 0.05) are indicated in bold text.

### Genome sequencing and mapping

The whole genome of the 47 S1 individuals of *M. larici-populina*, plus the parental strain 98AG31, was resequenced using the Illumina HiSeq 2000 technology. A total of over 40 billions bp were sequenced, with a relatively uneven repartition of sequencing depth per individual (Table S1). Over three quarters of reads (268 millions) mapped to the reference genome, leading to an average genome sequencing depth of 265X when considering all individuals, but ranging from 1.3X to 9.9X with most individuals presenting a final genome sequencing depth of about 5–7X. Genome sequences were deposited in GenBank under the BioProject ID PRJNA255081 and the SRA accession number SRP044324.

### Detection of SNPs and determination of recombination blocks

**Figure 3:**
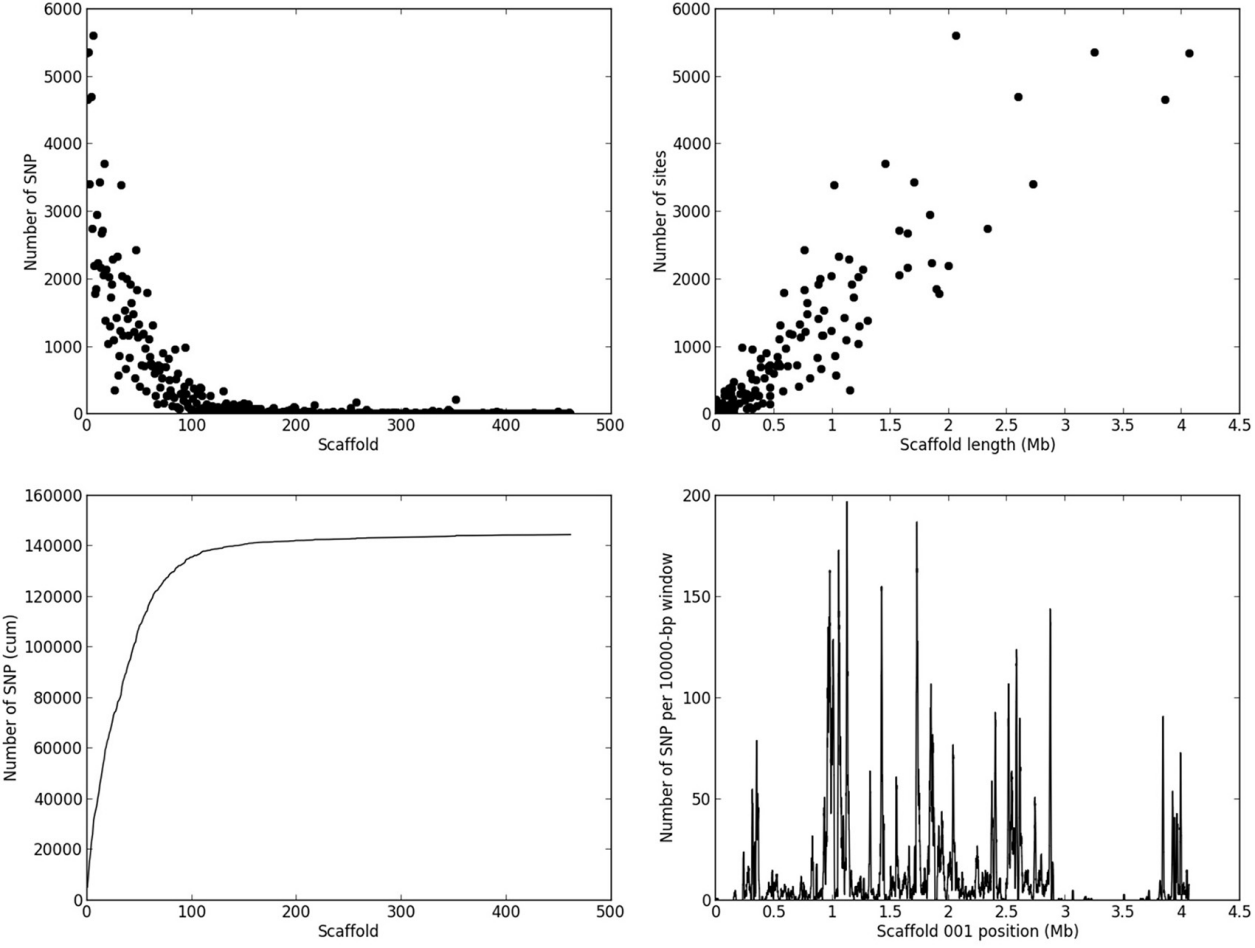
**Distribution of SNPs across the *M. larici-populina* genome**. The figure presents the number of SNPs detected per scaffold (upper-left panel, the scaffold are sorted by decreasing length in the genome assembly), and the resulting cumulative number of SNPs per scaffold (lower-left panel), the number of SNPs as a function of scaffold length (upper-right panel), and the local density of SNPs in scaffold 1 taken as an example (lower-right panel).

**Figure 4:**
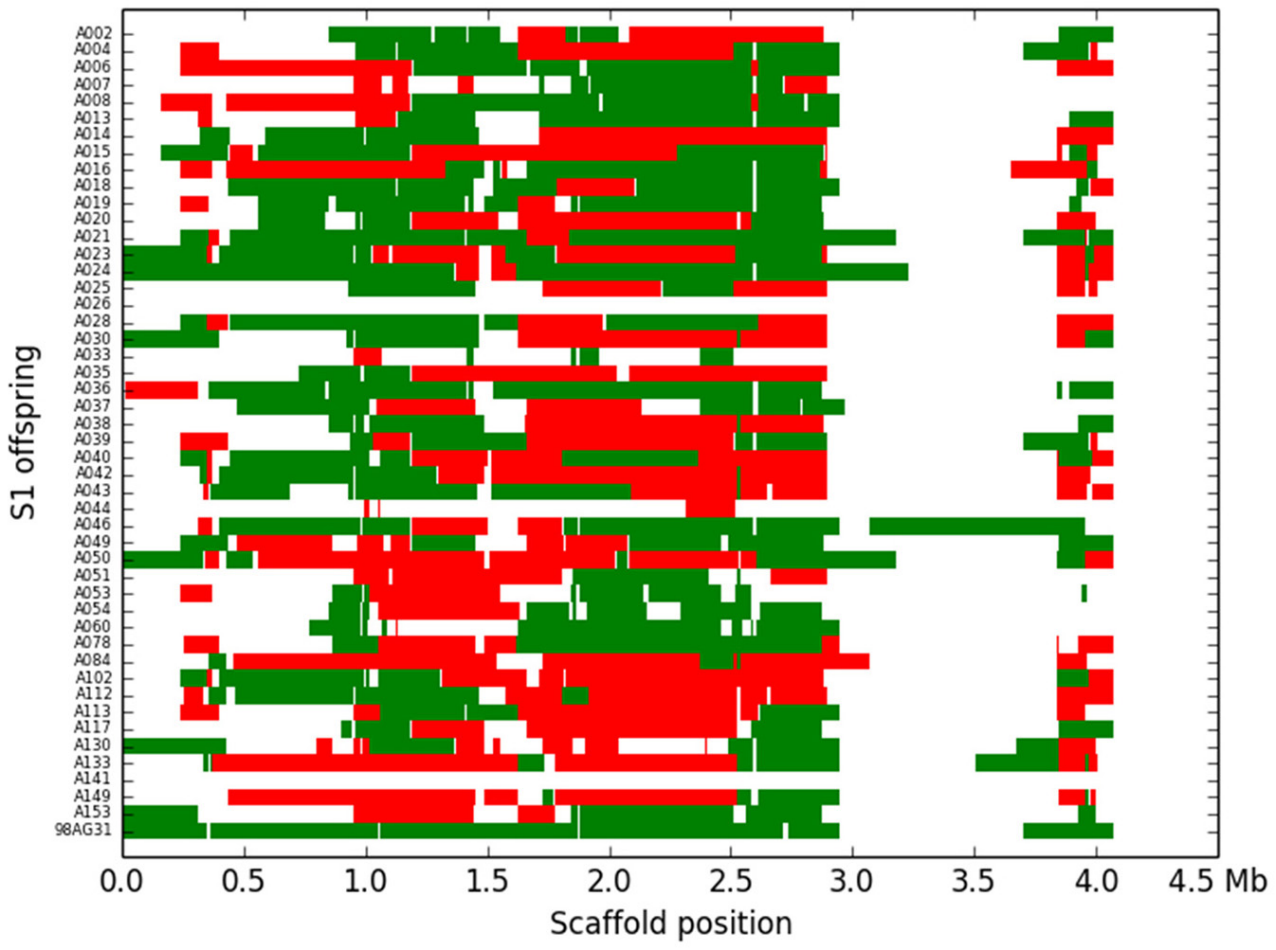
**Recombination blocks and assignment of the 48 *M. larici-populina* individuals (including the parental strain) for scaffold 1**. Each line represents an S1 individual, the last being the parental strain. The blocks are represented against their physical location along scaffold 1. Green blocks represent heterozygote regions, red blocks represent homozygote regions and white areas represent unassigned regions.

Based on the criteria described in Materials and Methods, we identified 144,566 SNPs across the genome. The average density is 1.4 SNP/kb, but exhibits a marked heterogeneity across the genome (see the example in Figure 3 for scaffold 1). Based on a smoothed scoring approach, we identified all potential recombination points and assigned regions to homozygote and heterozygote status (see the example in Figure 4 for scaffold 1). This led to the definition of a total of 3302 recombination blocks in the genome, of which 2580 exhibited two phylogenetically distinct homozygous alleles and were considered robust enough to be analyzed. It can be noted that the recombination blocks may have different sizes, both in terms of number of SNPs included and of physical region covered. The number of SNPs per block ranges from just 1 to 1465 and the length of the corresponding physical region reaches more than half a Mbp (Table S2). In total, 140 of the 462 scaffolds contain at least one block (other scaffolds either have no SNP or the recombination block was excluded). Sixty-one have more than 10 blocks, and the three largest scaffolds have over 100 blocks. Each individual appears to be a mixture of homozygous and heterozygous regions as expected, although there appears to be a slight bias toward homozygous regions (Table 3). As expected, the fraction of the genome of the parental strain that is assigned was exclusively heterozygous. For individuals for which sequencing depth was too low, the smoothed scores rarely achieved threshold scores and large fractions of their genome were left unassigned.

**Table 3:** **Proportion of recombination blocks determined as heterozygous, homozygous, or unassigned for all *M. larici-populina* individuals**.

**Individual**	**Homozygous**	**Heterozygous**	**Unassigned**
98AG31-A002	0.40	0.13	0.47
98AG31-A004	0.48	0.18	0.34
98AG31-A006	0.36	0.22	0.42
98AG31-A007	0.34	0.11	0.55
98AG31-A008	0.34	0.20	0.46
98AG31-A013	0.32	0.20	0.48
98AG31-A014	0.39	0.16	0.45
98AG31-A015	0.43	0.28	0.30
98AG31-A016	0.48	0.24	0.28
98AG31-A018	0.40	0.16	0.44
98AG31-A019	0.41	0.18	0.41
98AG31-A020	0.38	0.15	0.46
98AG31-A021	0.36	0.30	0.33
98AG31-A023	0.40	0.26	0.34
98AG31-A024	0.33	0.28	0.40
98AG31-A025	0.35	0.15	0.50
98AG31-A026	0.02	0.01	0.97
98AG31-A028	0.50	0.26	0.24
98AG31-A030	0.40	0.19	0.41
98AG31-A033	0.25	0.06	0.69
98AG31-A035	0.35	0.16	0.49
98AG31-A036	0.37	0.14	0.49
98AG31-A037	0.35	0.11	0.54
98AG31-A038	0.38	0.14	0.48
98AG31-A039	0.38	0.17	0.45
98AG31-A040	0.47	0.20	0.33
98AG31-A042	0.40	0.24	0.36
98AG31-A043	0.31	0.22	0.47
98AG31-A044	0.06	0.03	0.91
98AG31-A046	0.38	0.16	0.46
98AG31-A049	0.37	0.21	0.43
98AG31-A050	0.45	0.26	0.29
98AG31-A051	0.29	0.13	0.59
98AG31-A053	0.31	0.11	0.59
98AG31-A054	0.38	0.11	0.51
98AG31-A060	0.10	0.16	0.74
98AG31-A078	0.41	0.21	0.38
98AG31-A084	0.46	0.17	0.37
98AG31-A102	0.48	0.16	0.36
98AG31-A112	0.45	0.22	0.33
98AG31-A113	0.38	0.15	0.47
98AG31-A117	0.37	0.14	0.49
98AG31-A130	0.31	0.20	0.48
98AG31-A133	0.40	0.18	0.42
98AG31-A141	0.01	0.01	0.98
98AG31-A149	0.37	0.15	0.49
98AG31-A153	0.35	0.13	0.51
98AG31	0.00	0.27	0.73

### Analysis of genomic linkage disequilibrium

**Figure 5:**
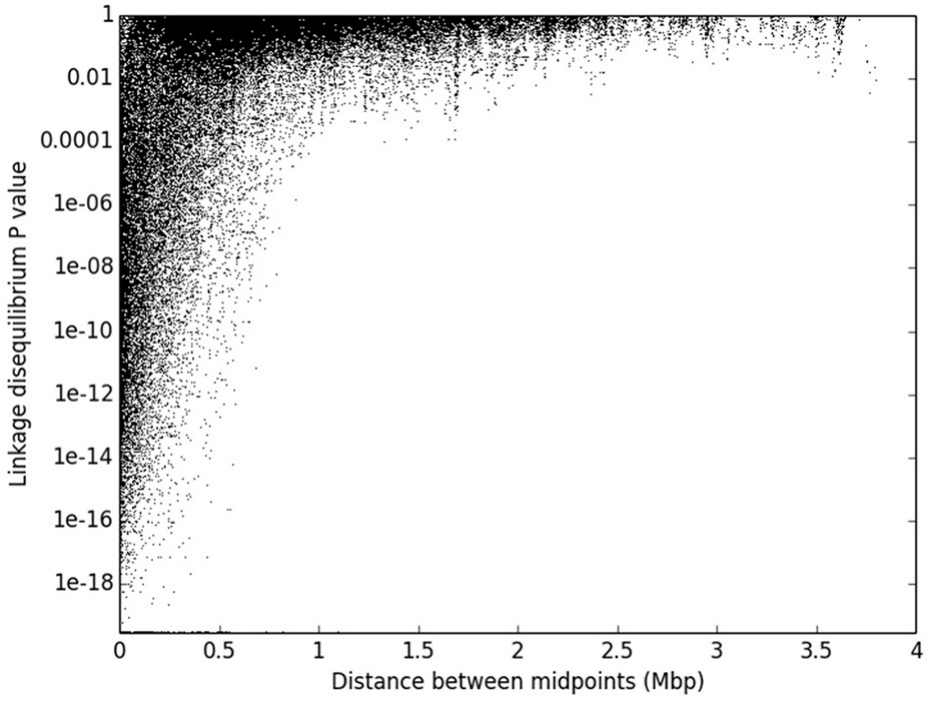
**Decay of linkage disequilibrium against physical distance**. The value of Fisher's exact test of association between 61,743 pairs of recombination blocks belonging to the same scaffold is represented as a function of the physical distance between block midpoints.

The 2580 recombination blocks were treated each as a diallelic marker with three genotypic states. All 3,326,910 pairwise comparisons were considered, and 3,149,154 Fisher's exact tests were performed (excluding pairs with too much missing data), of which 61,743 involved markers belonging to the same assembly scaffold. The analysis of the decay of linkage disequilibrium against physical distance shows that highly significant (*P*-values less than 10^−4^) linkage disequilibrium extends to over a distance of 1 Mbp (Figure 5).

**Figure 6:**
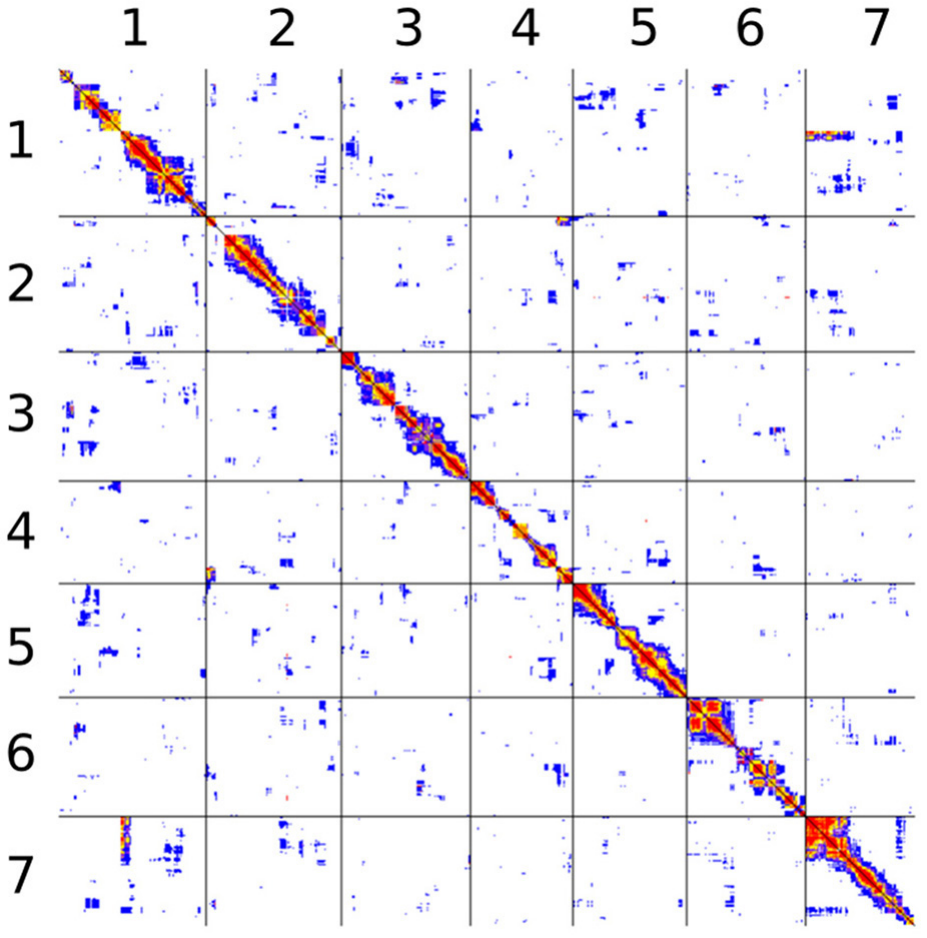
**Pairwise linkage disequilibrium between all markers belonging to assembly scaffolds 1 through 7**. The figure does not account for physical distance. Black lines separate scaffolds. All pairs are represented twice, above and below the diagonal. Each pair is represented by a single pixel. The color code is based on Fisher's exact test *P*-value: red, *P* < 10^−10^; orange, *P* < 10^−8^; yellow, *P* < 10^−6^; purple, *P* < 10^−4^; blue, *P* < 10^−2^; white, larger *P*-values or not computed.

The study of pairwise linkage disequilibrium test between all pairs of markers (both within and between assembly scaffolds) allowed us to identify signatures of statistical linkage between different scaffolds (see Figure 6 for a focus on the first 7 scaffolds). A systematic but liberal analysis identified 158 pairs of scaffolds that may follow each other, grouping a total of 81 scaffolds into 23 linkage groups (Table S3). Although 381 scaffolds (representing together over 35 Mbp) are left unlinked, the 81 grouped scaffolds contain 66 of the 100 largest scaffolds and represent more than 65 Mbp (65% of the genome). One of these links across scaffolds can be seen between scaffolds 2 and 4 in Figure 6 (second row, fourth column). While most pairs of sites exhibits low levels of linkage (white or blue pixels), a cluster of highly correlated pairs of sites appear at the upper-right corner of this panel, indicating linkage disequilibrium between the beginning of scaffold 2 and the end of scaffold 4.

However, this approach fails to account for signatures of linkage disequilibrium between scaffolds when they do not occur at the end of both scaffolds. A striking but not exceptional example can be seen in Figure 6 between the beginning of scaffold 7 and a region at the beginning of the second third of scaffold 1 (in the reverse orientation). In addition, there is no signature of linkage between the first and second thirds of scaffold 1. This pattern, which suggests that scaffolds are linked internally rather than serially, was found at multiple instances among pairwise comparisons, sometimes with internal connections in both scaffolds (Figure 6, Figure S1).

## Discussion

### Production of the *M. larici-populina* S1 progeny

In this study, we developed a segregating S1 progeny of the poplar rust fungus *M. larici-populina*, through selfing of the reference strain 98AG31 on larch plants. Pioneer work on classical genetics of rust fungi began in the 1930–1940's with the studies of Harold H. Flor on the flax/flax rust pathosystem (Flor, 1935, 1942). Since *Melampsora lini* is autoecious (i.e., completes its life cycle on a single host plant, flax), the production of F1 and F2 progenies through selfing or outcrossing was easier compared to heteroecious rusts. Nevertheless, early work on genetics of heteroecious cereal rusts also began in the 1940's (Johnson and Newton, 1946). In the present work several difficulties were overcome to manage the life cycle of the poplar rust fungus in controlled conditions. The first obstacle was the break of dormancy of teliospores. Several attempts were made to break dormancy in laboratory conditions with repeated cycles of freezing-thawing and wetting-drying for several months (Zambino et al., 2000), but remained unsuccessful. Thus, the “natural overwintering” method (Pei et al., 1999; Leonard and Szabo, 2005) was used instead.

The second difficulty stems from the fact that it is not possible to grow basidiospore-derived haploid mycelium of *M. larici-populina in vitro* in order to genotype a haploid progeny, as can be done with other Basidiomycetes, such as Agaricomycotina (Doudrick et al., 1995; Labbé et al., 2008), and with some rust fungi, such as the fusiform rust fungus (Doudrick et al., 1993). Therefore, we decided to inoculate larch plants, the alternate host, and to genotype aeciospore-derived dikaryotic individuals of *M. larici-populina*.

The third difficulty was the availability of fresh larch needles at just the time that germinating teliospores were available. Several attempts were made to infect detached larch flushing buds produced from 2-year-old larch plants (Pei et al., 1999), but with limited success because the larch needles could not be maintained alive for 2–3 weeks without rotting. The use of larch seedlings has overcome this difficulty.

The fourth difficulty was the feasibility of performing controlled outcrosses with *M. larici-populina* by matching larch needles bearing pycnia derived from different telial sources. Although some authors have obtained outcrossed progenies (Pei et al., 1999), it is technically difficult to obtain single isolated pycnia on larch needles in order to perform controlled spermatization of individual pycnia derived from one strain with spermatia derived from another strain. Therefore, we decided to study a S1 (selfed) progeny obtained through spermatization of pycnia from one telial source (strain 98AG31) with spermatia obtained from the same telial source. One drawback of this strategy is that only loci that are heterozygous in the parental strain segregate in the S1 progeny. Nevertheless, thanks to the low level of inbreeding found in natural *M. larici-populina* populations (Barrès et al., 2008; Xhaard et al., 2011, 2012), as many as 144,566 segregating SNPs were observed in the progeny studied, which is largely sufficient for mapping purposes.

### Segregation analysis

Results of the segregation analysis showed that two out of the 14 polymorphic microsatellite loci did significantly depart from the expected 1:2:1 ratio, namely loci MLP49 (located on scaffold 8) and MLP59 (located on scaffold 37). This result can still be explained by chance alone under Mendelian segregation. Applying the Bonferroni correction for multiple testing leads to a significance threshold of 0.0035, so that both loci no longer depart significantly from the expected ratio. Alternatively, a significant excess of heterozygotes may be due to the proximity of these loci to mating type loci. Eleven putative pheromone precursor genes and four pheromone receptor genes, which may be involved in the mating type, were annotated in the *M. larici-populina* genome (Duplessis et al., 2011). However, none of these potential mating type genes were located on scaffolds 8 or 37, where loci MLP49 and MLP59 are located. Considering that the genomic organization of the mating type loci is still unresolved for the poplar rust fungus, we cannot conclude that the mating type loci influence the excess of heterozygotes observed for loci MLP49 and MLP59.

Other forms of selection may cause an excess of heterozygotes, such as over dominant selection. This type of selection is known to occur within host-pathogen interactions (Hughes and Nei, 1988), and may therefore affect our experiment since *M. larici-populina* is an obligate biotroph and can be cultivated only in interaction with its host. Analysis of genome-wide data could help analyze putative signatures of selection. In this first study, however, SNPs obtained through whole-genome sequencing were filtered such as all three genotypes were represented, which could have caused a bias when testing for departure from Mendelian ratios, by excluding sites more often in case of distortion of Mendelian segregation.

### Linkage disequilibrium analyses

Pairwise linkage disequilibrium tests on microsatellite data allowed us to bring to light two pairs of microsatellite loci in genetic linkage. Three different cases were encountered: (i) loci known to be physically linked because they are located on the same scaffold and found genetically linked; (ii) loci that are not physically linked but found genetically linked; and (iii) loci that are not found genetically linked despite being physically linked.

As expected, loci MLP77 (scaffold 15; 629,856 bp) and MLP82 (scaffold 15; 1,199,792 bp) were found in significant linkage disequilibrium (*Q*-value < 10^−4^), since they are located on the same scaffold at 569,936 bp distance, which proved the efficiency of this method to confirm physical linkage by genetic linkage tests. In addition, locus MLP77 also was found in significant linkage disequilibrium with locus MLP56 (scaffold 5; 2,550,898 bp), with a *Q*-value < 10^−4^, despite located on different scaffolds. Consequently, this pair of loci is genetically linked and the respective scaffolds (scaffold 5 and scaffold 15) should be grouped into a linkage group. Furthermore, since locus MLP56 is located at the end of scaffold 5 and MLP77 is located at first third of scaffold 15, the most likely physical link should be between the end of scaffold 5 and the beginning of scaffold 15. Thus, the microsatellite-based linkage disequilibrium analysis allowed us to build one linkage group for a total length of 4,252,591 bp, which accounts for about 4.2% of the total genome length. However, these two scaffolds were not found in the same linkage group as defined from the SNP-based linkage analysis (Table S3).

Counter-intuitive results were observed for MLP94 (scaffold 1; 236,499 bp)/MLP55 (scaffold 1; 2,804,879 bp), and MLP54 (scaffold 8; 807,623 bp)/MLP59 (scaffold 8; 1,544,938 bp) pairs. Although each pair of loci is located on a single scaffold, no linkage disequilibrium was detected (*Q*-value = 0.77 and 0.74, respectively). Loci MLP94 and MLP55 are located on scaffold 1 at a distance of 2,568,380 bp, which could be far enough to break any genetic linkage. This result is consistent with the SNP-based linkage analysis, which showed that linkage is unlikely to be detected at distances higher than 2 Mbp. However, the situation is different for loci MLP54 and MLP59, which are located on scaffold 8 at a distance of 737,315 bp, and for which no linkage disequilibrium was found despite this relatively small distance. The SNP-based linkage analysis showed that linkage could be still detectable at distances up to 1 Mbp. This loss of linkage disequilibrium could be due to a recombination hotspot in this specific region (Petes, 2001). Another possible explanation would be misassembly of scaffold 8, resulting in a chimeric scaffold. This latter hypothesis is supported by the linkage disequilibrium discontinuities observed along scaffold 8 (Figure S1).

In a second step, we aimed to adopt a whole-genome perspective on linkage disequilibrium patterns. We used shifts from homozygous to heterozygous genomic regions detected from whole-genome sequencing data to scale genomic data down to 2580 recombination blocks in which all SNPs were non-recombining. These blocks were then treated as markers, allowing us to integrate data from successive SNPs, and to cope with sequencing errors and missing data that could have had a strong impact due to the relatively small sample size and the unequal sequencing depth among individuals. The approach was however limited to genomic regions that were polymorphic within the parental strain, thereby generating informative segregating markers in the offspring. Thanks to the low level of inbreeding and the relatively high level of polymorphism in the *M. larici-populina* populations, the amount of diversity proved to be sufficient for our purpose (more than one SNP per kb on average).

Linkage disequilibrium could be detected between markers located at nearby locations on the same assembly scaffold, but also between markers located on different scaffolds, while many pairs of markers located on the same scaffold exhibited no linkage disequilibrium. We found that, as expected, linkage disequilibrium decays with increasing distance, as shown with microsatellite markers cited earlier, with linkage still detectable at distances up to 1 Mbp. Based on the 2580 SNP-based markers, we detected 23 putative linkage groups, including scaffolds that exhibit signals of linkage disequilibrium with at least one other scaffold of the same group. Interestingly, some of the between-scaffold signatures of linkage disequilibrium obtained with whole-genome sequencing data pointed out potential genome assembly issues. As tentatively evidenced using microsatellite-based analysis, it appears that, at several instances, contiguous genomic regions within a scaffold display discontinuities in genetic linkage. These results point to the fact that most of the largest scaffolds might have to be redefined, i.e., split and rearranged. Due to the large size and high repetitive sequence content (for a fungus), the genome of *M. larici-populina* is not easy to assemble properly, even based on high-depth genome sequencing. It is thus possible that some of the scaffolds are actually chimers.

Noteworthy, the pattern of linkage disequilibrium among these whole-genome markers (23 linkage groups) did not match the observation made using the 14 microsatellite markers. In other words, the linkage group made of scaffold 5 and 15 evidenced by the microsatellite-based linkage analysis is not supported by the SNP-based scaffold merging. This discrepancy can be further explained by a careful look at the pattern of SNP-based linkage along scaffold 15 (Figure S1) and may constitute another example of putative genome misassembly. First, we observed a clear break in statistical linkage between the very beginning of scaffold 15 and the rest of this scaffold. Second, while the very beginning of scaffold 15 is undoubtedly linked with scaffold 12 (which defined linkage group 7, Table S3), we observed that the SNP-based markers located just after the genetic break (beginning of the second part of scaffold 15) are statistically linked with markers located at the end of scaffold 5. This observation is fully consistent with the linkage group formed by the microsatellite loci MLP77 (scaffold 15) and MLP56 (scaffold 5). The microsatellite-based analysis thus enables us to extend the linkage group 4 (consisting in scaffold 73 linked to the beginning of scaffold 5) by adding the largest portion of scaffold 15 (linked with the end of scaffold 5).

The linkage disequilibrium analysis performed over a narrow number (14) of microsatellite markers allowed us to define one linkage group in the *M. larici-populina* genome. The subsequent use of 2480 markers integrating the information of almost 150,000 SNPs detected in 47 S1 individuals suggested 23 linkage groups. Both methods have strengths and weaknesses—microsatellites were typed on more offspring individuals, and SNPs are available at higher density along genomes—but they are complementary. While the SNP-based and microsatellite-based linkage analyses appeared inconsistent at first sight, we demonstrated that these two analyses converged to question current genome assembly. These encouraging results demonstrate that an accurate genetic map could be constructed with a larger number of *M. larici-populina* S1 offspring, by using the method for constructing ultra-high-density linkage maps with high-quality SNPs based on low-coverage resequencing, as already described for plants (Huang et al., 2009; Xie et al., 2010) and fungi (Au et al., 2013).

Besides constituting by itself a valuable resource for investigating the architecture of complex traits of *M. larici-populina*, such a genetic map will provide complementary data for completing the genome assembly. The assembly of the genome of *M. larici-populina* has been made difficult by its large size (compared to most fungi) and especially its high content in repetitive sequences (Duplessis et al., 2011). However, the reference genome is an essential resource for genetic approaches as genes of interest might be much more difficult to identify if they are located in repeat-rich and poorly assembled regions. Genetic mapping is an excellent complementary approach, because, unlike physical sequencing, it is much less sensitive to the presence of repetitive sequences (it still is because of the possibility of ambiguous mapping). The integration of genetic mapping data to the current version of the genome assembly of *M. larici-populina* might therefore represent a significant advance.

## Author contribution

Michaël Pernaci, Stéphane De Mita, Fabien Halkett, and Pascal Frey designed research; Michaël Pernaci, Stéphane De Mita, Axelle Andrieux, Jérémy Pétrowski, and Pascal Frey performed research; Michaël Pernaci, Stéphane De Mita, Fabien Halkett, and Pascal Frey analyzed data; and Michaël Pernaci, Stéphane De Mita, Fabien Halkett, Sébastien Duplessis, and Pascal Frey wrote the paper.

### Conflict of interest statement

The authors declare that the research was conducted in the absence of any commercial or financial relationships that could be construed as a potential conflict of interest.
