# Supplementary material for: Genome-wide patterns of segregation and linkage disequilibrium: the construction of a linkage genetic map of the poplar rust fungus Melampsora larici-populina
Source: Front Plant Sci. 2014 Sep 10;5:454. doi: 10.3389/fpls.2014.00454 (PMC4159982; doi:10.3389/fpls.2014.00454)
Supplement: Supplementary file 1 [file DataSheet1.PDF]

Supplementary Material

Genome-wide patterns of segregation and linkage disequilibrium: the construction of a linkage genetic map of the poplar rust fungus *Melampsora larici-populina*

Michaël Pernaci[1,2], Stéphane De Mita[1,2], Axelle Andrieux[1,2], Jérémy Pétrowski[1,2], Fabien Halkett[1,2], Sébastien Duplessis[1,2], Pascal Frey[1,2]*

[1]INRA, Interactions Arbres - Microorganismes, UMR1136, Champenoux, France.

[2]Université de Lorraine, Interactions Arbres - Microorganismes, UMR1136, Vandoeuvre-lès-Nancy, France.

***Correspondence**:
Dr. Pascal Frey
INRA
Interactions Arbres – Microorganismes, UMR1136
F-54280 Champenoux, France
pascal.frey@nancy.inra.fr

Supplementary Tables

Supplementary Table 1. Sequencing and mapping statistics for the 47 *M. larici-populina* S1 individuals and the parental strain.

Individual	Sequenced reads	Sequenced bp	Mapped reads	Genome coverage depth	Mapped rate
98AG31-A002	6,537,882	653,788,200	5,511,182	5.4X	84%
98AG31-A004	7,633,030	763,303,000	7,221,125	7.1X	95%
98AG31-A006	8,390,738	839,073,800	7,736,359	7.6X	92%
98AG31-A007	6,485,602	648,560,200	5,733,613	5.7X	88%
98AG31-A008	8,703,514	870,351,400	6,917,456	6.8X	79%
98AG31-A013	7,739,638	773,963,800	6,237,001	6.2X	81%
98AG31-A014	6,868,808	686,880,800	6,409,755	6.3X	93%
98AG31-A015	4,996,024	499,602,400	4,564,972	4.5X	91%
98AG31-A016	4,259,856	425,985,600	3,878,620	3.8X	91%
98AG31-A018	7,308,296	730,829,600	6,079,167	6.0X	83%
98AG31-A019	5,268,304	526,830,400	5,029,899	5.0X	95%
98AG31-A020	6,025,534	602,553,400	5,368,402	5.3X	89%
98AG31-A021	5,400,744	540,074,400	5,070,312	5.0X	94%
98AG31-A023	4,041,120	404,112,000	3,674,820	3.6X	91%
98AG31-A024	5,388,224	538,822,400	4,866,207	4.8X	90%
98AG31-A025	5,805,818	580,581,800	5,455,985	5.4X	94%
98AG31-A026	2,767,773	276,777,300	2,616,181	2.6X	95%
98AG31-A028	4,803,744	480,374,400	4,413,313	4.3X	92%
98AG31-A030	7,268,514	726,851,400	6,448,081	6.4X	89%
98AG31-A033	7,427,998	742,799,800	3,523,515	3.5X	47%
98AG31-A035	6,353,702	635,370,200	5,076,956	5.0X	80%
98AG31-A036	5,141,152	514,115,200	4,481,271	4.4X	87%
98AG31-A037	6,584,058	658,405,800	4,469,952	4.4X	68%
98AG31-A038	6,370,410	637,041,000	5,322,772	5.3X	84%
98AG31-A039	9,006,302	900,630,200	6,576,558	6.5X	73%
98AG31-A040	9,394,234	939,423,400	8,379,544	8.3X	89%
98AG31-A042	8,113,708	811,370,800	7,616,109	7.5X	94%
98AG31-A043	10,894,066	1,089,406,600	8,917,167	8.8X	82%
98AG31-A044	4,027,101	402,710,100	1,619,076	1.6X	40%
98AG31-A046	7,928,318	792,831,800	5,979,766	5.9X	75%
98AG31-A049	10,001,042	1,000,104,200	8,241,776	8.1X	82%
98AG31-A050	5,374,076	537,407,600	3,283,859	3.2X	61%
98AG31-A051	6,110,100	611,010,000	4,265,151	4.2X	70%
98AG31-A053	7,347,692	734,769,200	4,982,745	4.9X	68%
98AG31-A054	6,841,020	684,102,000	5,013,145	5.0X	73%
98AG31-A060	5,917,374	591,737,400	5,424,026	5.4X	92%
98AG31-A078	4,590,944	459,094,400	4,107,538	4.1X	89%
98AG31-A084	5,910,098	591,009,800	5,438,376	5.4X	92%
98AG31-A102	6,055,696	605,569,600	5,742,311	5.7X	95%
98AG31-A112	9,716,540	971,654,000	8,749,793	8.7X	90%
98AG31-A113	5,776,378	577,637,800	5,184,218	5.1X	90%
98AG31-A117	40,870,838	4,087,083,800	5,912,239	5.8X	14%
98AG31-A130	10,526,694	1,052,669,400	9,983,737	9.9X	95%
98AG31-A133	7,228,226	722,822,600	6,845,384	6.8X	95%
98AG31-A141	1,754,919	175,491,900	1,308,002	1.3X	75%
98AG31-A149	6,190,676	619,067,600	5,356,466	5.3X	87%
98AG31-A153	6,955,960	695,596,000	6,605,620	6.5X	95%
98AG31	7,731,436	773,143,600	7,004,617	6.9X	91%
Total	351,833,921	35,183,392,100	268,644,139	265.6X	76%

Supplementary Table 2. Statistics of recombination blocks identified in all scaffolds of the *M. larici-populina* genome.

Scaffold #	Blocks	Min SNPs	Max SNPs	Average SNPs	Min bp	Max bp	Average bp
1	115	1	756	45.1	1	333797	27033.5
2	106	1	375	41.2	1	382810	30816.5
3	101	1	824	49.7	1	213884	24585.8
4	80	1	400	39.3	1	413923	27013.5
5	89	1	421	51.6	1	438478	27107.2
6	93	1	369	27.2	1	537303	19922.8
7	85	1	932	64.2	1	237008	22721.7
8	56	1	340	38.1	1	299332	31920.4
9	50	1	208	28.6	1	157933	18675.6
10	50	1	259	35.7	1	359872	35832.6
11	57	1	460	49.7	1	501196	26699.5
12	50	1	589	42.5	1	420660	35521.2
13	56	1	829	60.8	1	218874	29072.3
14	31	1	367	63.4	1	299450	48191.1
15	53	1	592	45.7	1	284041	23343.6
16	51	1	450	51.0	1	204235	28282.2
17	44	1	590	42.5	1	158136	19304.7
18	73	1	660	47.1	1	141062	15625.5
19	21	1	723	62.6	1	264082	42948.1
20	41	1	397	50.3	1	122090	26951.3
21	8	1	634	130.4	1	396459	150942.6
22	43	1	320	45.8	1	421946	25835.7
23	35	1	222	36.4	1	502478	34103.6
24	27	1	326	57.7	1	319743	35257.0
25	38	1	378	46.9	1	347314	29661.3
26	31	1	515	72.6	1	216218	34968.9
27	27	1	345	35.9	1	429815	36354.6
28	4	3	148	64.3	1095	420920	109996.5
29	27	1	284	48.2	1	509700	34455.3
30	37	1	505	60.0	1	131191	25379.8
31	2	2	307	154.5	1633	215792	108712.5
32	7	16	475	122.0	20612	518820	142404.0
33	28	1	213	40.9	1	122745	24994.8
34	55	1	478	57.9	1	105455	13700.5
35	38	1	409	42.4	1	109748	17743.8
36	14	1	481	80.6	1	340902	55621.0
37	25	1	337	53.6	1	138573	22260.9
38	14	1	158	31.6	1	244698	33286.6
39	55	1	489	34.3	1	221625	14044.6
40	26	1	349	52.5	1	177739	29701.1
41	24	2	216	45.4	49	172684	31883.1
42	15	1	342	55.1	1	265967	55119.8
43	47	1	286	33.0	1	77821	12505.2
44	39	1	467	40.3	1	163085	18310.7
45	16	1	529	86.3	1	163179	41744.5
46	9	1	535	110.8	1	179521	36454.1
47	7	9	117	37.4	5001	74628	41394.4
48	6	4	1465	399.3	4819	316842	121843.2
49	43	1	194	42.6	1	126878	16626.6
50	21	1	316	51.6	1	165006	32879.8
51	22	1	712	60.0	1	284682	32367.2
52	7	3	101	50.9	202	137320	34148.9

53	12	4	168	50.4	6082	119608	37039.6
54	32	1	238	35.2	1	147563	17161.9
55	31	1	326	38.0	1	147165	19669.5
56	3	12	528	208.0	1496	505158	204804.3
57	17	1	263	56.0	1	197245	33636.2
58	1	145	145	145.0	187724	187724	187724.0
59	27	1	873	66.1	1	194622	20620.1
60	18	1	435	59.2	1	312475	29759.9
61	13	3	271	59.1	61	112933	32712.1
62	15	1	201	38.1	1	197573	20606.0
63	4	28	361	175.8	1846	309042	126774.3
64	16	1	417	74.3	1	300614	31834.1
65	17	1	220	31.5	1	41255	8452.7
66	2	268	326	297.0	197804	301045	249424.5
67	3	48	141	79.0	16594	58675	43039.7
68	1	147	147	147.0	463446	463446	463446.0
69	21	1	218	33.8	1	150723	21287.1
70	18	1	88	16.2	1	30404	3780.1
71	4	8	169	82.0	4231	326904	109349.3
72	13	1	226	53.2	1	199263	34307.5
73	5	1	340	100.8	1	184247	66377.2
74	5	6	474	131.2	16590	76097	35597.0
76	12	1	225	51.6	1	93131	26940.8
77	7	1	215	37.7	1	325405	50988.4
78	8	1	69	20.0	1	74121	14613.1
79	2	48	766	407.0	55093	329568	192330.5
80	1	494	494	494.0	340243	340243	340243.0
81	8	1	133	42.0	1	206155	40336.5
82	10	1	95	27.9	1	101308	30538.1
84	2	85	147	116.0	135651	152064	143857.5
85	6	29	434	158.8	2315	105197	46257.0
86	6	1	159	49.3	1	159734	31652.3
87	3	11	50	34.3	1163	121148	48092.7
88	16	1	151	32.8	1	80553	16905.9
91	7	1	135	41.9	1	87578	27120.3
92	11	1	144	27.6	1	146188	21476.4
94	1	403	403	403.0	218141	218141	218141.0
95	12	1	314	81.9	1	55219	18277.2
96	6	1	123	45.8	1	145912	34482.3
97	1	151	151	151.0	203401	203401	203401.0
98	2	43	57	50.0	47590	110414	79002.0
99	1	468	468	468.0	158783	158783	158783.0
102	10	1	56	20.3	1	40508	11989.5
103	4	52	140	94.8	15889	63376	33244.3
106	1	152	152	152.0	117407	117407	117407.0
107	4	1	211	54.3	1	61884	16062.8
110	2	151	227	189.0	43567	58129	50848.0
111	9	1	142	30.3	1	76977	11054.2
112	1	92	92	92.0	107445	107445	107445.0
115	1	70	70	70.0	41065	41065	41065.0
117	1	73	73	73.0	89146	89146	89146.0
119	2	51	213	132.0	13075	65036	39055.5
121	1	3	3	3.0	8458	8458	8458.0
122	1	59	59	59.0	58830	58830	58830.0
126	1	41	41	41.0	36854	36854	36854.0
131	1	335	335	335.0	72575	72575	72575.0
134	4	3	35	13.8	301	33207	9839.5
136	1	86	86	86.0	57077	57077	57077.0

141	1	45	45	45.0	34542	34542	34542.0
142	1	52	52	52.0	59929	59929	59929.0
148	1	123	123	123.0	57297	57297	57297.0
149	1	70	70	70.0	52310	52310	52310.0
150	1	57	57	57.0	27796	27796	27796.0
151	1	66	66	66.0	36584	36584	36584.0
152	1	72	72	72.0	40992	40992	40992.0
155	1	84	84	84.0	39343	39343	39343.0
156	1	147	147	147.0	22622	22622	22622.0
158	1	28	28	28.0	24235	24235	24235.0
161	1	80	80	80.0	36944	36944	36944.0
164	1	69	69	69.0	40983	40983	40983.0
168	1	76	76	76.0	31962	31962	31962.0
169	1	21	21	21.0	10955	10955	10955.0
180	1	85	85	85.0	18940	18940	18940.0
197	1	45	45	45.0	8221	8221	8221.0
200	1	59	59	59.0	16776	16776	16776.0
204	1	101	101	101.0	11750	11750	11750.0
205	2	38	43	40.5	1319	12947	7133.0
215	1	49	49	49.0	14594	14594	14594.0
219	1	27	27	27.0	12389	12389	12389.0
225	1	38	38	38.0	4182	4182	4182.0
226	6	2	63	22.5	18	9600	2214.2
263	1	82	82	82.0	9997	9997	9997.0
268	1	167	167	167.0	5873	5873	5873.0
282	1	65	65	65.0	7828	7828	7828.0
360	1	24	24	24.0	6935	6935	6935.0
381	1	57	57	57.0	8656	8656	8656.0
390	2	66	152	109.0	1829	4503	3166.0
555	1	31	31	31.0	2282	2282	2282.0
Total	2580	1	1465	50.6	1	537303	28885.7

Supplementary Table 3. Candidate linkage groups in the *M. larici-populina* genome detected through between-scaffold linkage disequilibrium.

Linkage group	Number of scaffolds	Total length	Scaffolds (unordered)
1	2	4,384,908	001, 085
2	9	11,256,268	002, 004, 014, 022, 044, 059, 080, 161, 180
3	7	6,377,598	003, 026, 046, 050, 086, 110, 156
4	2	3,027,187	005, 073
5	2	2,547,235	006, 096
6	10	6,805,533	010, 028, 039, 043, 049, 067, 088, 095, 103, 148
7	2	3,509,521	012, 015
8	2	2,933,134	013, 021
9	3	2,593,998	017, 063, 070
10	2	1,516,315	018, 164
11	3	2,237,450	019, 064, 077
12	3	2,766,535	020, 033, 066
13	9	4,817,406	024, 047, 056, 060, 072, 079, 081, 092, 094
14	2	1,697,133	025, 065
15	4	1,624,364	035, 062, 131, 226
16	2	910,612	038, 390
17	2	1,178,973	040, 087
18	5	2,461,657	041, 054, 061, 091, 121
19	2	797,037	052, 119
20	2	605,644	071, 106
21	2	591,141	076, 097
22	2	495,527	078, 107
23	2	305,619	099, 102
unlinked	381	35,688,233	All others
