# Supplementary material for: Genome-wide patterns of segregation and linkage disequilibrium: the construction of a linkage genetic map of the poplar rust fungus Melampsora larici-populina
Source: Front Plant Sci. 2014 Sep 10;5:454. doi: 10.3389/fpls.2014.00454 (PMC4159982; doi:10.3389/fpls.2014.00454)

Supplementary Figure S1:

Pairwise linkage disequilibrium between all markers belonging to assembly scaffolds 5 through 15. The figure does not account for physical distance. Black lines separate scaffolds. All pairs are represented twice, above and below the diagonal. Each pair is represented by a single pixel. The color code is based on Fisher's exact test *P*-value: red, $P < 10^{-10}$; orange, $P < 10^{-8}$; yellow, $P < 10^{-6}$; purple, $P < 10^{-4}$; blue, $P < 10^{-2}$; white, larger *P*-values or not computed.

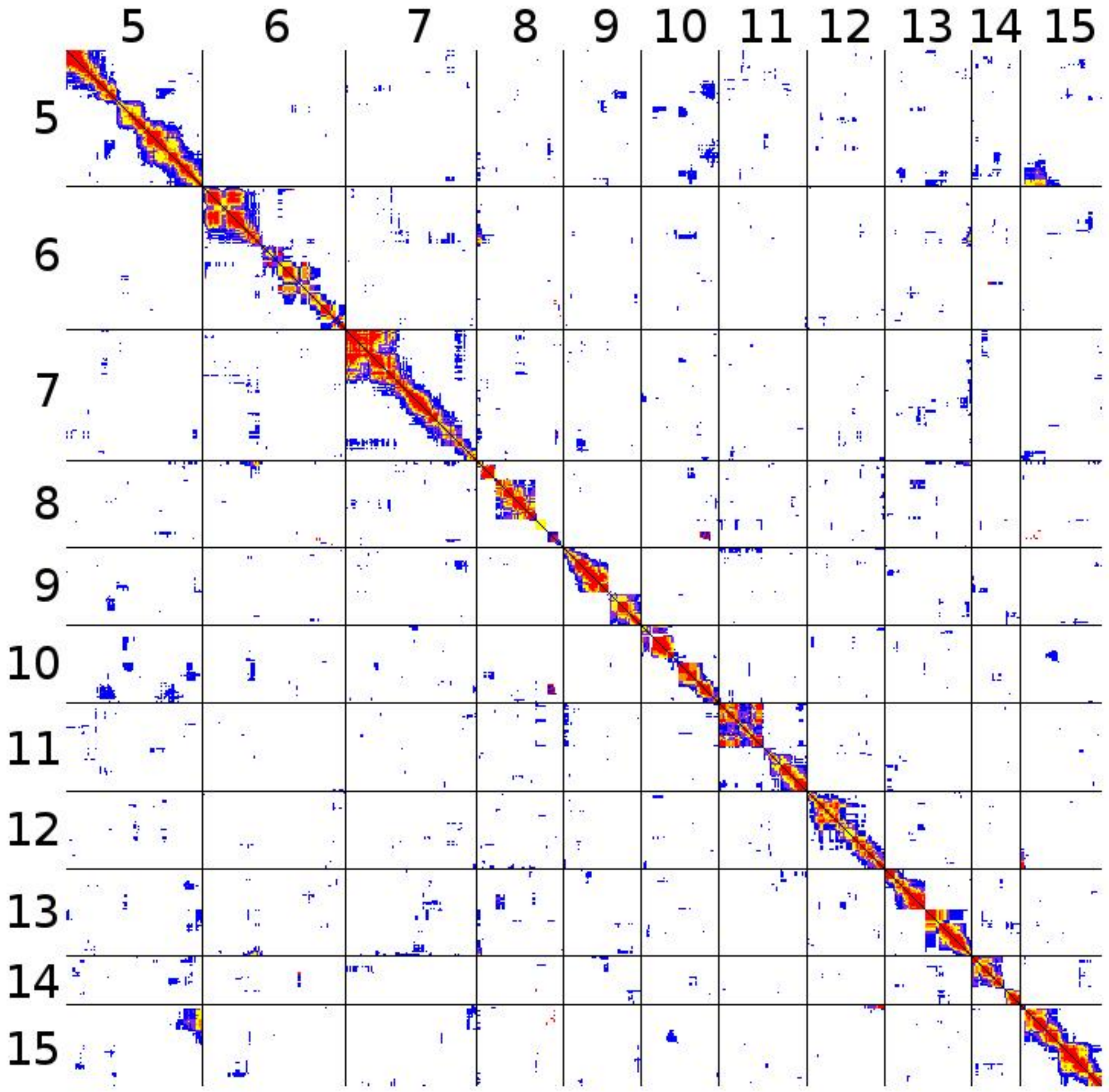

Supplement: Supplementary file 2 [file Image1.PDF]
